# Reassessing and Extending the European Standards of Care for Newborn Health: How to Keep Reference Standards in Line with Current Evidence

**DOI:** 10.3390/children11020179

**Published:** 2024-02-01

**Authors:** Isabel Geiger, Johanna Kostenzer, Valerie Matthäus, on behalf of the ESCNH Chair Committee, Silke Mader, Luc J. I. Zimmermann

**Affiliations:** 1 European Foundation for the Care of Newborn Infants (EFCNI), 81379 Munich, Germany; isabel.geiger@efcni.org (I.G.); johanna.kostenzer@efcni.org (J.K.); valerie.matthaeus@efcni.org (V.M.); luc.zimmermann@efcni.org (L.J.I.Z.); 2 Department of Paediatrics, Research School for Oncology and Reproduction (GROW), Maastricht UMC+, 6229 HX Maastricht, The Netherlands

**Keywords:** European collaboration, standards of care, newborn health, improvement of care, open consultation

## Abstract

The European Standards of Care for Newborn Health (ESCNH) were launched in 2018. After three years, the first standards were reassessed and revised to align with current evidence. Moreover, new standards regarding emerging topics were developed. The aim of this paper is to outline the approach adopted for reassessing, revising and developing new standards for the ESCNH. We established a systematic approach to reassess the ESCNH including a public and an expert consultation. The public consultation was open to all stakeholders for feedback whereas the expert consultation followed a targeted consultation method. For developing new standards, a similar process to the original development was implemented. Overall, 20 standards were reassessed and six standards were developed. For the revision process, 23 experts were involved in the targeted consultation method and 253 questionnaires were completed via the open consultation. We demonstrated a systematic approach to update and extend reference standards, which can be applied by other developers of standards. Thereby, we highlighted that including a public and an expert consultation is crucial to improve quality and to ensure that all stakeholder perspectives are integrated.

## 1. Introduction

The European Standards of Care for Newborn Health (ESCNH), a set of 96 standards, were officially launched at the European Parliament in Brussels in November 2018. The aim of the ESCNH is to establish a common framework to improve care for hospitalised newborn infants on a European level [1]. The ESCNH are not legally binding but are intended as a starting point for building binding country-specific guidelines, regulations or laws. After three years, 20 out of 96 standards reached their predefined first revision cycle as part of an overall lifecycle approach, where standards are reassessed after a timespan of either three or five years. This revision process, together with the process of developing new standards, will be described in this paper.

The ESCNH were initiated by the European Foundation for the Care of Newborn Infants (EFCNI). Together with a group of about 220 international and multidisciplinary experts including parent/patient representatives, eleven comprehensive topics of newborn health were defined, where the development of standards of care was considered particularly important. This multidisciplinary approach is aiming to guarantee that all stakeholder perspectives are acknowledged and integrated under the guidance of a Chair Committee, which serves as steering body of the ESCNH, each topic was led and further developed by a chair team. More information regarding the composition and role of the Chair Committee, the chair team and other stakeholders as well as regarding the general development of the ESCNH can be found in the published project protocol [1].

Ever since their launch, there have been several initiatives to promote the implementation of the ESCNH in order to improve the quality of care for preterm infants throughout Europe. For example, in Italy, a group of highly motivated healthcare professionals formed an implementation taskforce. In coordination with the Italian parent organisation, they translated the ESCNH into Italian and distributed the translated version to all NICUs in the country to make the ESCNH more accessible to users. Based on the hands-on and clear format of the standard template, action points per standard can be directly applied by the respective user group (e.g., neonatal units or healthcare professionals) [1].

Reference standards such as the ESCNH require regular updates to ensure that they align with the newest evidence [2]. Systematic approaches to review or update clinical guidelines or standards of care are of great importance to assure high quality and safety [3]. National institutions such as the National Institute for Health and Care Excellence (NICE) in the UK or the Australian National Health and Medical Research Council (NHMRC) have published handbooks for quality standard and guideline development [4,5,6]. One vital aspect when developing or updating guidelines and standards is to engage and work with patients and the public. In this respect, the Guidelines International Network (GIN) issued a toolkit for guideline developers on involving patients and the public [7]. Despite the available tools for developing or updating guidelines [8], there is no uniformly applicable process and thus the update is strongly dependent on the responsible organisation. Contemporary literature focusses mainly on the development and update of clinical guidelines [3,5,6,7,8,9] and few references were found on standards of care and quality [2,4]. We therefore integrated literature on both to define our methodology.

The aim of this paper is to transparently report the comprehensive methodological approaches used for reassessing and extending standards of care, using the ESCNH as an example. Our focus is improving quality of newborn care and ensuring coherence of evidence. Additionally, we present and reflect on the results obtained during the reassessment and extension process to optimise future processes, which will bring added value to scholars, healthcare managers and professionals who study, develop, revise or wish to use reference standards.

## 2. Materials and Methods

**Figure 1:**
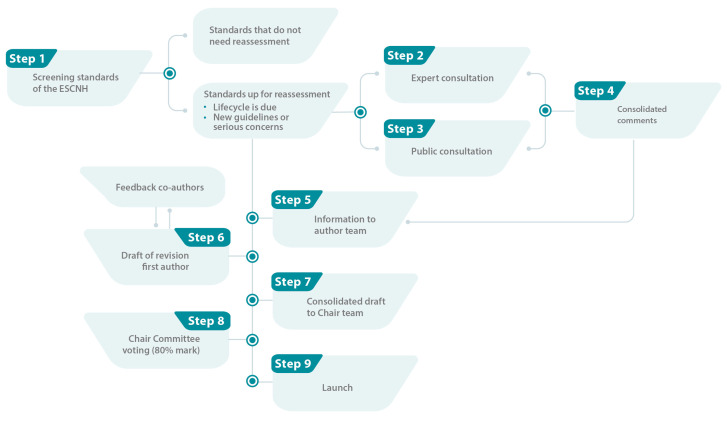
Simplified approach for revising the European Standards of Care for Newborn Health (ESCNH, own illustration).

**Figure 2:**
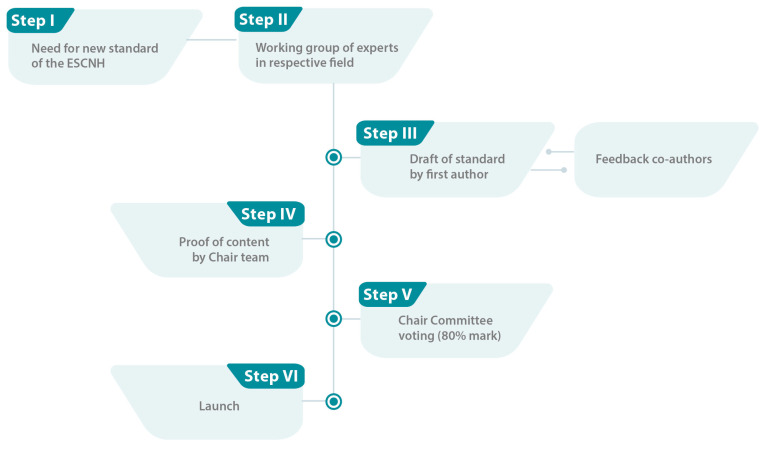
Simplified approach for developing new standards for the European Standards of Care for Newborn Health (ESCNH, own illustration).

To revise the ESCNH, we developed a 9-step approach (Figure 1). To develop new standards, we have adapted the original ESCNH development process (Figure 2).

### 2.1. Revision Process

The revision process was coordinated by the respective Project Manager (PM) at EFCNI, EFCNI’s Medical Director (MD) and the Chairwoman of EFCNI’s Executive Board (CW), who are subsequently referred to as the ESCNH team.

In Step 1, the PM together with the MD identified which standards needed to be reassessed based on their predefined lifecycle. They also screened systematically whether any other standards required a revision based on newly available evidence or major concerns expressed previously by healthcare professionals, members of the Chair Committee or parent/patient representatives.

In Step 2, the ESCNH team set up an expert consultation by adopting a targeted consultation method to ensure conclusiveness, reliability, and timeliness of evidence [6]. Key opinion leaders in the respective medical field were identified through extensive literature searches per topic and recommendations by international renowned experts, and they were invited to volunteer as independent reviewers of a standard. All selection criteria for the reviewers can be found in Table 1. In total, up to two experts per standard were requested to review the standard via an online survey using the SurveyMonkey^®^ online tool [10]. The survey consisted of one question per section of the standard in addition to further questions regarding the quality, applicability and legitimacy of the recommendations provided in the standard (a template of the survey can be found in Supplementary File S1).

Simultaneously to the expert consultation, the ESCNH team also set up a public consultation (Step 3) by combining open and targeted consultation approaches using an online survey [5]. Email invitations were sent to all partners and supporters of the ESCNH including parent/patient representatives of national parent organisations (*n* = 51), national and international health professional associations (*n* = 124), authors of standards not under reassessment (*n* = 112) and industry partners (*n* = 5). All recipients were asked to share the email with their network or other relevant stakeholders. Additionally, the public consultation was promoted via EFCNI’s social media channels (LinkedIn, Facebook, Twitter, Instagram), website and newsletter to ensure that the wider community had the opportunity to contribute. For reasons of transparency, all comments from the public consultation submitted via the online survey were published after the revised standards were launched on the ESCNH website. Respondents could request that their contribution should not be published, when stating ample justification. In accordance with the GDPR, the organisations of the respondents are displayed only if they approved this in the survey.

After the consultations were closed, all feedback was consolidated by the PM and MD (Step 4).

In Step 5, the author teams of the standards received the consolidated comments of both consultations. The first author of each standard was then asked to review the comments and revise the standard in agreement with the co-authors. The first authors were not obliged to include any comment obtained by the consultations if the requested changes were not in line with current evidence. However, the first authors (in cooperation with the co-authors) had to give reason why the comment is not scientifically sound.

After a revised draft was available and confirmed by all co-authors (Step 6), the respective chair team of the topic the standard belongs to was asked to review the content (as an additional measure for quality control) and subsequently approve the draft (Step 7). Once the content of the standard was definite, the ESCNH team edited the standard in an identical manner as during the original development process [1].

In case of major changes in the text compared to the original version of the standard, which was evaluated by the respective chair team together with the ESCNH team, the standard required a new vote by the Chair Committee (Step 8) in accordance with the original developing process. Each standard needed to be reconfirmed in an online voting by an 80% majority [1]. If a standard did not pass the 80% acceptance mark, it had to be adapted by the responsible working group, re-starting the process from Step 6.

Finally, all standards that passed the voting or had minor revisions were officially re-launched (Step 9).

### 2.2. Development of New Standards

Following the original development process of the ESCNH [1], new interdisciplinary working groups were established to draft standards which were identified as emerging subjects within the framework of the eleven overarching topics. The need for a standard in these areas was established through expert opinion and supporting evidence from literature (Step I).

Once a leader of the working group was identified based on track record and competence in the topic to be addressed, and willingness to contribute (first author), other key opinion leaders were invited to join the working group (Step II). Identical to the set-up of previous working groups, at least one parent/patient representative was involved in the development of a new standard. Additionally, industry partners in related medical fields were able to delegate one medical expert per organisation to join the working group as silent observers.

After several online meetings of the working group, the first author was responsible for developing a first draft of the standard with the co-authors (Step III).

When a final draft was available and agreed upon by all co-authors, the respective chair team was asked to review the content and provide approval (Step IV). Once the content of the new standard was definite, the ESCNH team edited the standard in an identical manner as during the original development process [1].

All newly developed standards required a vote by the Chair Committee (Step V) in accordance with the original developing process before being launched (Step VI). Each standard had to be confirmed in an online voting by an 80% majority to be accepted [1]. If the voting was not passed, the responsible working group had to start again at Step III.

Further details on the original process are available in the project protocol and on the ESCNH website [1,11].

## 3. Results

### 3.1. Revision Process

The revision process started in June 2021 and lasted over 16 months. The systematic screening of the ESCNH identified 13 standards, due for reassessment in 2021 based on their original lifecycle. Additionally, seven standards required a revision based on newly available evidence (e.g., the new European Resuscitation Council Guideline 2021 [12]) or major concerns expressed previously by healthcare professionals. A list of all 20 standards reassessed in 2021 can be found in Table 2.

#### 3.1.1. Expert Consultation

After a thorough literature search by the ESCNH team and exchanges with suitable candidates, 23 independent medical experts were identified to each systematically assess one of the standards through an online survey (see Table 2). The majority of standards (*n* = 17) was reviewed by one independent reviewer, whereas three standards were assessed by two independent reviewers. Despite efforts to maintain gender equality, most experts identified as male (*n* = 15) and, overall, had a median age of 45–54 years (*n* = 11). Their professions ranged from neonatologists, neonatal nurses and paediatricians to anaesthesiologists, psychologists and ophthalmologists depending on the respective topic of the standard.

#### 3.1.2. Public Consultation

In the public consultation, 63 individuals took part (Table 2). The standard which was examined by the highest number of respondents (*n* = 19) was hypoglycaemia in at-risk term infants in the ’Medical care and clinical practice’ topic and the standard with the lowest turnout (*n* = 8) was respiratory outcome in the ’Follow-up and continuing care’ topic. The majority of respondents expressed wanting to improve the care of newborn infants (77.8%) and/or working in the respective medical field (55.5%) as one of their reasons for participation. A further 14.3% of respondents were parents of a preterm or ill newborn infant. Responses were received from 24 countries, with most participants originating from the Czech Republic. In contrast to the expert consultation, the majority of respondents identified as female (69.4%) and had a median age of 55–64 years. The anonymised results of the public consultation were published on the ESCNH website [11]. Organisations were mentioned, if permission was given to do so.

Both consultations were open from the beginning of August 2021 until mid-October 2021 and were followed by the revision process through the author teams, which ran between November 2021 and April 2022.

#### 3.1.3. Quality Rating

Respondents of both consultations (expert and public) were asked to rate the quality of the respective standards on a scale from one to five, with five being the highest and one being the lowest rating possible. The experts rated 15 standards with a quality rating of four or above; the remaining five standards—hypoglycaemia in at risk term infants, neurological monitoring in the high-risk infant (near-infrared spectroscopy), taking blood samples, effective implementation of early parenteral feeding and prevention of necrotising enterocolitis—received quality ratings of three or a combination of three and four, in the case of neurological monitoring in the high-risk infant (near-infrared spectroscopy). When being asked whether the experts would recommend the reviewed standard for use, 86.3% (*n* = 19) confirmed the use of the standard either in its current form (*n* = 14) or with indicated modifications (*n* = 5). Three standards were not recommended for use by one respondent each (prevention of bronchopulmonary dysplasia, taking blood samples and prevention of necrotising enterocolitis). However, the experts provided suggestions on how to improve their quality, which were considered in the revised versions.

**Figure 3:**
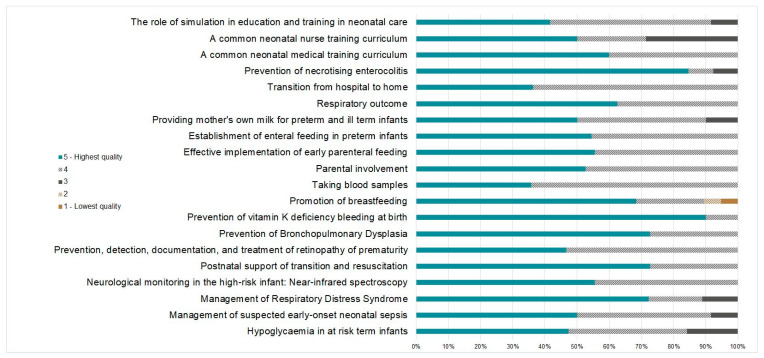
Simplified approach for developing new standards for the European Standards of Care for Newborn Health (ESCNH, own illustration).

As shown in Figure 3, the public consultation produced similar results: apart from the standards hypoglycaemia in at-risk term infants (84%), management of respiratory distress syndrome (89%), promotion of breastfeeding (89%), and a common neonatal nurse training curriculum (71%), all standards were rated by at least 90% of respondents with a quality rating of four and above. Promotion of breastfeeding was rated with low scores of one and two, respectively, by two respondents.

### 3.2. Development of New Standards

In total, six new standards were developed following the original development process over the course of two years, starting in 2021 [1,11]. The working groups varied in size between 6 and 11 experts and the standards covered the following subjects: cord management at the delivery of preterm and cord management at the delivery of term infants (both in the topic ’Birth and transfer’); diagnosis and management of necrotising enterocolitis (’Medical care and clinical practice’); immunisation of preterm infants (’Follow-up and continuing care’); threat, error and success reporting (how to effectively practice error management) (’Patient safety and hygiene practice’); and quality indicators (’Data collection and documentation’).

All meetings were held online. No silent observers were delegated by industry partners to any working group meetings of the newly developed standards.

### 3.3. Voting Process and Launch

After the revision process was finalised and the editing of the reassessed standards was completed, the respective chair teams together with the ESCNH team identified eight standards which were classified as requiring major revisions and thus needed a new vote by the Chair Committee (hypoglycaemia in at risk term infants, management of suspected early-onset neonatal sepsis, prevention of bronchopulmonary dysplasia, parental involvement, effective implementation of early parenteral feeding, establishment of enteral feeding in preterm infants, providing mother’s own milk for preterm and ill term infants, prevention of necrotising enterocolitis).

Based on the original development process of the ESCNH, all new standards required a vote by the Chair Committee, which was held together with majorly revised standards. The vote was conducted via an online survey, during five weeks from 21 June 2022. In total, 27 out of 35 (77%) Chair Committee members participated in the voting. All standards passed the 80% mark for approval, with percentages varying from 84.6% (parental involvement) up to 100% (hypoglycaemia in at risk term infants) and a mean 93.6% approval rate. Reasons obtaining answer categories ‘no’ and ‘abstain’ will be shared with the author teams for the next reassessment of the respective standards.

In September 2022, the revised and newly developed standards were jointly launched and promoted at medical congresses [13,14]. All revised and updated standards are available on the ESCNH website [11].

Due to changes in the first authors of the standards as well as staff shortages due to the COVID-19 pandemic, three standards could not be finalised and are expected to be voted on and launched in the second quarter of 2023 (promotion of breastfeeding, taking blood samples and the prevention of vitamin K deficiency bleeding at birth).

## 4. Discussion

In this paper, we report the systematic and comprehensive methodology used for the reassessment of standards on the newest empirical evidence and the development process of new standards of the European Standards of Care for Newborn Health (ESCNH). By applying a combination of targeted and open consultation methods in public and expert consultations, we were able to reach a diverse portfolio of respondents to improve quality and increase further the general acceptance of all 20 revised standards. Additionally, new working groups were set up to extend the ESCNH by six standards that were not part of the original set.

The results of both consultations show that the overall quality of the ESCNH was perceived as high, even before the reassessment and extension. Standards that were rated with lower quality scores or which were not recommended for use by the reviewing expert received a major revision by the author teams. This highlights the importance of external consultations before revising a standard to further improve quality and to facilitate the revision process by revealing areas that need adaptation.

We decided to use individualised surveys for both consultations (see Supplementary File S1) instead of applying published appraisal tools for guidelines such as ‘The Appraisal of Guidelines for REsearch & Evaluation (AGREE)’ instrument [15] as we did not deem their questions suitable to our purposes. Since AGREE was designed, amongst others, for guideline developers “to follow a structured and rigorous development methodology, to conduct an internal assessment to ensure that their guidelines are sound, or to evaluate guidelines from other groups for potential adaptation to their own context” and for healthcare professionals “who wish to undertake their own assessment of a guideline before adopting its recommendations into their practice”, the underlying statements are of a general nature and not targeted enough to the unique template of the ESCNH, especially with regard to the independent expert consultation, where each section of the standard was appraised. Many questions of the tool would have been already fulfilled because the standard template is designed to highlight, e.g., the population to whom it is meant to apply (target group). However, to ensure uniformity to other public consultation approaches, we consulted the surveys for public consultations of standards used by other developers of standards, e.g., NICE [4], when developing our survey. The future development of an evaluated appraisal tool for standards would be desirable for international comparison and consistency.

We were unable to obtain detailed information in the literature regarding the duration of the consultation when more than one standard or guideline is being reviewed. Most studies, however, suggest a general consultation period of at least four weeks [4,5,7], with GIN highlighting that there is no best practice example in this regard [7]. In an attempt to balance updating the standards in a timely fashion while considering the public’s and other stakeholder’s expectations and after seeking the Chair Committee of the ESCNH’s expert opinion, we set the consultation period to 10 weeks. No additional focus groups were set up as the discussion with the Chair Committee already indicated that there is no internationally available consensus/knowledge to draw from. Ten weeks, however, was deemed as a reasonable time frame to ensure that all stakeholders have the opportunity to take part in the consultation. Reminders sent via email to healthcare associations and parent organisations as well as social media promotion were used to ensure continuous submissions of consultation surveys throughout the entire consultation period.

The individual links to the public consultation surveys per standard were listed consecutively per topic and in alphabetical order on the ESCNH website [11]. After evaluating the results, a small trend (R^2^ = 0.1324) could be identified regarding the order of the surveys and the number of respondents: namely, the further down the list of standards, the less respondents were recorded. This phenomenon is not new, as also other scholars reported on the impact of, e.g., question order on the results [16,17]. However, in our case, as the number of standards was relatively limited (*n* = 20), the trend was negligible as standards which were listed 9th or 11th were answered by 19 and 18 respondents, respectively. To avoid potential biases towards the number of respondents in future, especially if the number of standards under revision rises, all standards that are open for public consultation need to be displayed in an equally accessible manner on the website. One approach could be to shuffle the order after a certain time (e.g., one week) in order to increase the likelihood of a standard to be assessed.

The original development process of the ESCNH included many in-person meetings, which were time-consuming and costly as experts involved in the process came from across Europe and beyond. Due to the outbreak of the COVID-19 pandemic, the new standards were developed during online meetings, which turned out to be similarly productive. Moreover, more experts were able to attend meetings on a regular basis as they could join the meeting directly from their workplaces. To maintain efficiency and cost-effectiveness, it appears preferable to develop future standards also primarily in online sessions, holding additional in-person meetings only in specific cases.

### Strengths and Limitations

Our methodology for reassessing and extending the ESCNH combines current evidence for guideline and standard development and update. The existing expertise of the author groups of each standard was enlarged by a comprehensive peer consultation in the form of both the independent expert and the open public consultation. Through the integration of feedback from healthcare professionals and other stakeholders like parent organisations, we aimed at achieving the best possible quality. Despite being tailored to fit to the ESCNH, our methodology can be adapted and applied by other scholars, healthcare managers or professionals who are working with reference standards and are interested in quality improvement.

One limitation of the public consultation concerns the use of our official ESCNH supporters list to send out invitation emails, which consists of healthcare societies, parent organisations and industry partners. Although the ESCNH have a great number of partners and supporters, organisations which are currently not supporting the ESCNH might not have been reached successfully over other channels like social media and, thus, might be underrepresented in the review of the standards.

Another aspect with regard to the public consultation concerns the number of submitted surveys per person. Five respondents submitted surveys for all 20 standards, thus representing almost 40% of the overall number of surveys. Despite the fact that respondents who filled in all surveys did not give feedback on how to improve the standard, it has to be acknowledged that their opinions (especially regarding their quality rating) might have been overrepresented in the results. However, as the majority of respondents submitted only one or two standards and as standards received on average feedback from seven additional individuals, we believe the feedback to be balanced. Future consultations could limit the number of surveys submitted per person to ensure that a single person’s opinion is not overrepresented in the results.

## 5. Conclusions

This paper describes our systematic approach to update and extend reference standards for medical care, which may be applied by other standard developers. By including both a public and an expert consultation, we ensured that not only healthcare professionals’ views were integrated in the revision but offered also parents and the greater public the chance to shape medical care directly.

## Figures and Tables

**Table 1:** Selection criteria for the independent expert consultation.

Criteria	Justification
**Inclusion**	
Proven track record in respective field	Experts are invited only if they have a proven track record in terms of recent publications or other relevant activities in the respective field.
Employment based in Europe	As the ESCNH are tailored for the European region, experts invited need to work primarily in Europe.
**Exclusion**	
Previous involvement in the ESCNH	To ensure independence, experts who were involved in the initial development of the standards are excluded.
Conflict of interest	Experts that work exclusively for related industries such as pharmaceutical companies or present any other major conflict of interest are excluded from expert consultation which is determined by the ESCNH team.

**Table 2:** Overview of findings from the public and expert consultation. Responses marked with (*) were not requested from experts, (**) refers to questions where multiple answers were possible, (^T^) indicates that one consultation form was filled in by two individuals and (°) refers to standards that were reassessed based on newly available evidence or major concerns expressed previously by healthcare professionals.

Category	Characteristics	Expert Consultation	Public Consultation	Total
Respondents	Total	*n* = 22 (+1 ^T^)	*n* = 63	*n* = 85
Age	25–34	*n* = 1	*n* = 3	*n* = 4
	35–44	*n* = 3	*n* = 9	*n* = 12
	45–54	*n* = 11	*n* = 15	*n* = 26
	55–64	*n* = 6	*n* = 22	*n* = 28
	65–74	*n* = 0	*n* = 9	*n* = 9
	Over 74	*n* = 0	*n* = 2	*n* = 2
	Missing response	*n* = 1	*n* = 3	*n* = 4
Gender	Female	*n* = 6	*n* = 43	*n* = 49
	Male	*n* = 15	*n* = 18	*n* = 33
	Missing response	*n* = 1	*n* = 2	*n* = 3
Interest in consultation **	Parent of a preterm/ill newborn infants	N/A *	*n* = 9	N/A *
	Improve the care of newborn infants.		*n* = 49	
	Work in the respective medical field.		*n* = 35	
	Work in the respective industry.		*n* = 5	
	Other interests.		*n* = 11	
Country of residence	Belgium, Bulgaria, Croatia, Cyprus, Finland, Greece, Hungary, Ireland, Kosovo, Lithuania, Romania, Slovakia and Ukraine	N/A *	*n* = 1	N/A *
	Australia, Spain, Poland		*n* = 2	
	Portugal		*n* = 3	
	The Netherlands, United Kingdom		*n* = 4	
	Germany, United States of America		*n* = 5	
	Estonia		*n* = 6	
	Italy		*n* = 8	
	Czech Republic		*n* = 9	
Conflicts of interest **	Similar research projects	*n* = 2	*n* = 0	*n* = 2
	Member of medical society on respective topic	*n* = 1	*n* = 0	*n* = 1
	Grants/sponsorship received from industry	*n* = 4	*n* = 1	*n* = 5
	Working for industry	*n* = 1	*n* = 3	*n* = 4
Surveys completed	Total	*n* = 22(+1 ^T^)	*n* = 253	*n* = 275 (+1 ^T^)
Surveys submitted per standard	Hypoglycaemia in at risk term infants	*n* = 1	*n* = 19	*n* = 20
	Management of Respiratory Distress Syndrome °	*n* = 1	*n* = 18	*n* = 19
	Management of suspected early-onset neonatal sepsis	*n* = 1	*n* = 11	*n* = 12
	Neurological monitoring in the high-risk infant: Near-infrared spectroscopy °	*n* = 2	*n* = 9	*n* = 11
	Postnatal support of transition and resuscitation °	*n* = 1	*n* = 12	*n* = 13
	Prevention, detection, documentation, and treatment of ROP	*n* = 1	*n* = 15	*n* = 16
	Prevention of Bronchopulmonary Dysplasia	*n* = 1	*n* = 11	*n* = 12
	Prevention of vitamin K deficiency bleeding at birth °	*n* = 1	*n* = 10	*n* = 11
	Promotion of breastfeeding °	*n* = 1	*n* = 19	*n* = 20
	Taking blood samples °	*n* = 1	*n* = 14	*n* = 15
	Parental involvement	*n* = 1	*n* = 18	*n* = 19
	Effective implementation of early parenteral feeding	*n* = 1	*n* = 9	*n* = 10
	Establishment of enteral feeding in preterm infants	*n* = 1	*n* = 11	*n* = 12
	Providing mother’s own milk for preterm and ill term infants °	*n* = 1	*n* = 10	*n* = 11
	Respiratory outcome	*n* = 1 (+1 ^T^)	*n* = 8	*n* = 9
	Transition from hospital to home	*n* = 1	*n* = 11	*n* = 12
	Prevention of necrotising enterocolitis	*n* = 1	*n* = 13	*n* = 14
	A common neonatal medical training curriculum	*n* = 1	*n* = 10	*n* = 11
	A common neonatal nurse training curriculum	*n* = 1	*n* = 14	*n* = 15
	The role of simulation in education and training in neonatal care	*n* = 2	*n* = 11	*n* = 13
Surveys submitted per person	1 survey	*n* = 23	*n* = 29	*n* = 52
	2 surveys	*n* = 0	*n* = 14	*n* = 14
	3 surveys	*n* = 0	*n* = 5	*n* = 5
	4 surveys	*n* = 0	*n* = 4	*n* = 4
	5 surveys	*n* = 0	*n* = 3	*n* = 3
	14 surveys	*n* = 0	*n* = 1	*n* = 1
	17 surveys	*n* = 0	*n* = 1	*n* = 1
	19 surveys	*n* = 0	*n* = 1	*n* = 1
	20 surveys	*n* = 0	*n* = 5	*n* = 5

## Data Availability

The data presented in this study are available on request from the corresponding author. The data are not publicly available due to privacy reasons.

## Supplementary Materials

The following supporting information can be downloaded at: https://www.mdpi.com/2227-9067/11/2/179/s1, Supplementary File S1: consultation questionnaires.
