# Correction: Geiger et al. Reassessing and Extending the European Standards of Care for Newborn Health: How to Keep Reference Standards in Line with Current Evidence. *Children* 2024, *11*, 179

**DOI:** 10.3390/children11060653

**Published:** 2024-05-28

**Authors:** Isabel Geiger, Johanna Kostenzer, Valerie Matthäus, on behalf of the ESCNH Chair Committee, Silke Mader, Luc J. I. Zimmermann

**Affiliations:** 1 European Foundation for the Care of Newborn Infants (EFCNI), 81379 Munich, Germany; isabel.geiger@efcni.org (I.G.); valerie.matthaeus@efcni.org (V.M.); luc.zimmermann@efcni.org (L.J.I.Z.); 2 Department of Paediatrics, Research School for Oncology and Reproduction (GROW), Maastricht UMC+, 6229 HX Maastricht, The Netherlands

## Missing Institutional Review Board Statement

In the original publication [1], the Institutional Review Board Statement was not mentioned: No ethical approval was required for the study in accordance with the requirements of ethical committees such as the medical University of Munich (LMU) (https://www.med.lmu.de/einrichtungen/ethik/beratungspflicht/index.html (accessed on 14 December 2023)) and the Bavarian ethical committee (https://ethikkommission.blaek.de/studien/sonstige-studien/antragsunterlagen-ek-primarberatend-15-bo (accessed on 14 December 2023)) as the data of the online survey was anonymised for the analysis and neither sensitive nor person related data was processed. All data was processed in strict complaints with GDPR requirements. This correction was approved by the Academic Editor. The original publication has also been updated.
