# Supplementary material for: Reassessing and Extending the European Standards of Care for Newborn Health: How to Keep Reference Standards in Line with Current Evidence
Source: Children (Basel). 2024 Feb 1;11(2):179. doi: 10.3390/children11020179 (PMC10887008; doi:10.3390/children11020179)
Supplement: Supplementary file 1 [file children-11-00179-s001.zip › children-2778085-supplementary.pdf]

Public consultation

Questions

1. How would you rate the overall quality of the standard "[insert name of the standard]"?
2. Do you have any recommendations on how to improve the standard "[insert name of the standard]" (e.g. rationale, benefits, components)?
3. Is there any important evidence or useful guidelines you would recommend to be included in the standard "[insert name of the standard]"?
4. Do you think the standard "[insert name of the standard]" is relevant in your country?
5. *Optional*: Would you be willing to promote the implementation of the standard "[insert name of the standard]" or the ESCNH in general (e.g. share it with your organisation/community)?

Personal information

6. Do you have any conflict of interest (e.g. financial support or consulting of industry)?
7. Please specify if you are responding as an individual or on behalf of an organisation.
8. Personal details: Name, organisation, country, email
9. Why are you interested in the revision of the ESCNH?
10. Have you directly participated in any processes to formally endorse the ESCNH?
11. I hereby confirm that my responses to questions 1-4 and 6 can be published (displaying my organisation) after the revision on the ESCNH website.
12. I hereby confirm that my name (no email address) can be listed next to my responses to questions 1-4 and 6 after the revision on the ESCNH website.
13. Are we allowed to contact you via email in case there are any queries concerning your feedback?
14. Data protection policy

The following questions are for statistical purpose only (not mandatory).

- What is your age?
- What is your gender?

Expert consultation

Questions

1. How would you rate the overall **quality** of the standard "[insert name of the standard]"?
2. Please give your **main reason(s)** for the quality-rating above.
3. Are you aware of any **important evidence** that should be included in the standard "[insert name of the standard]"?
4. Is there any evidence to **invalidate** any of the recommendations comprising the standard "[insert name of the standard]"?
5. Do you agree with the **population** to whom the standard "[insert name of the standard]" is meant to apply ("target group" and "user group")?
6. Do you agree with the **statement of the standard** "[insert name of the standard]"?
7. Do you agree with the **rationale** of the standard "[insert name of the standard]"?
8. Do you agree with the **long-term** and **short-term benefits** of the standard "[insert name of the standard]"?
9. Do you agree with the **components** of the standard "[insert name of the standard]"?
10. Do you agree with the **recommendations** of the standard "[insert name of the standard]" ("getting started" and "where to go")?
11. Do you agree with the **description** of the standard "[insert name of the standard]"?
12. Would you **recommend** the standard "[insert name of the standard]" for use?

Personal information

13. Do you have any conflict of interest (e.g. financial support or consulting of industry)?
14. Personal details: Name, profession, country, email
15. I hereby confirm that I can be named as reviewer after the revision on the ESCNH website.
16. Data protection policy

The following questions are for statistical purpose only (not mandatory).

17. What is your age?
18. What is your gender?
